# Unique Dermoscopic and Histopathologic Features of Melanoma Arising in a Patient With BAP1 Tumor Predisposition Syndrome

**DOI:** 10.7759/cureus.17485

**Published:** 2021-08-27

**Authors:** Eric A Grisham, Joseph Tadros, Emily Smith

**Affiliations:** 1 Dermatology, University of Missouri, Columbia, USA; 2 Internal Medicine, Providence Sacred Heart, Spokane, USA

**Keywords:** melanoma, dermoscopy, hereditary, genetic skin disorders, brca 1 gene, brca associated protein 1 tumor predisposition syndrome, brca associated protein 1, tumor predisposition syndrome

## Abstract

Breast cancer gene 1 (*BRCA1*)-Associated Protein Tumor Predisposition Syndrome (BAP1-TPDS) is a relatively newly discovered syndrome that may develop a variety of malignancies, including atypical melanoma resembling Spitz nevi. Dermoscopic and molecular findings aid in diagnosing melanoma in BAP1-TPDS, and clinicians should have a high index of suspicion and a low threshold for screening and diagnostic testing for cutaneous malignancies in these patients. We describe an atypical, amelanotic melanoma in a 45-year-old male with a history of BAP1-TPDS and nodular melanoma. The patient presented with a rapidly evolving lesion on the right arm. Given the patient's prior history of melanoma and of heterozygous *BAP1* gene mutation, histopathological and molecular analysis was performed on the lesion, revealing a diffuse loss of *BAP1 *expression and multiple chromosomal aberrancies. In these cases, histopathological and molecular analysis remain the keys to diagnosis, but astute dermoscopic evaluation may help clinicians avoid initial diagnostic confusion and delays in treatment. Melanoma should always be considered in patients with BAP1-TPDS, even in the absence of classic gross, dermoscopic, histopathological, and molecular characteristics of typical melanoma.

## Introduction

Breast cancer (BRCA)-Associated-Protein-1 (BAP1) tumor predisposition syndrome (BAP1-TPDS) is a syndrome associated with an increased risk of cutaneous, uveal, and visceral malignancies. The *BAP1* gene was initially described by Harbour et al. in the context of uveal melanoma in 2010 [1]. Though some mutations in *BAP1* are sporadic, patients with biallelic and heritable *BAP1* mutations lack the function necessary to regulate transcription, differentiation, and DNA damage response through the ataxia-telangiectasia (ATM) pathway [2]. We are reporting a case of melanoma in a 45-year-old male with BAP1-TPDS. To our knowledge, this is the first description of both the dermoscopic and the histopathologic findings associated with a unique presentation of melanoma within a BAP1-inactivated tumor.

## Case presentation

**Figure 1:**
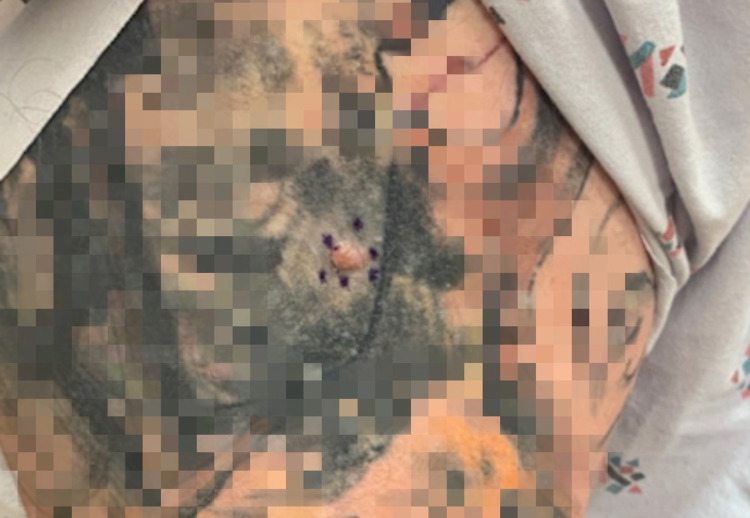
Clinical visualization of a suspicious, 7 mm, flesh-colored, firm papule adjacent to but not overlying a tattoo.

A 45-year-old male with a history of BAP1-TPDS manifesting as basal cell carcinoma, renal cell carcinoma, and cutaneous melanoma with a documented heterozygous mutation of the *BAP1* gene presented for surveillance examination [3]. A 7 millimeter (mm), flesh-colored, firm, recently enlarging papule was noted on the patient’s right upper extremity proximal to but not overlying a fully healed tattoo (Figure 1).

**Figure 2:**
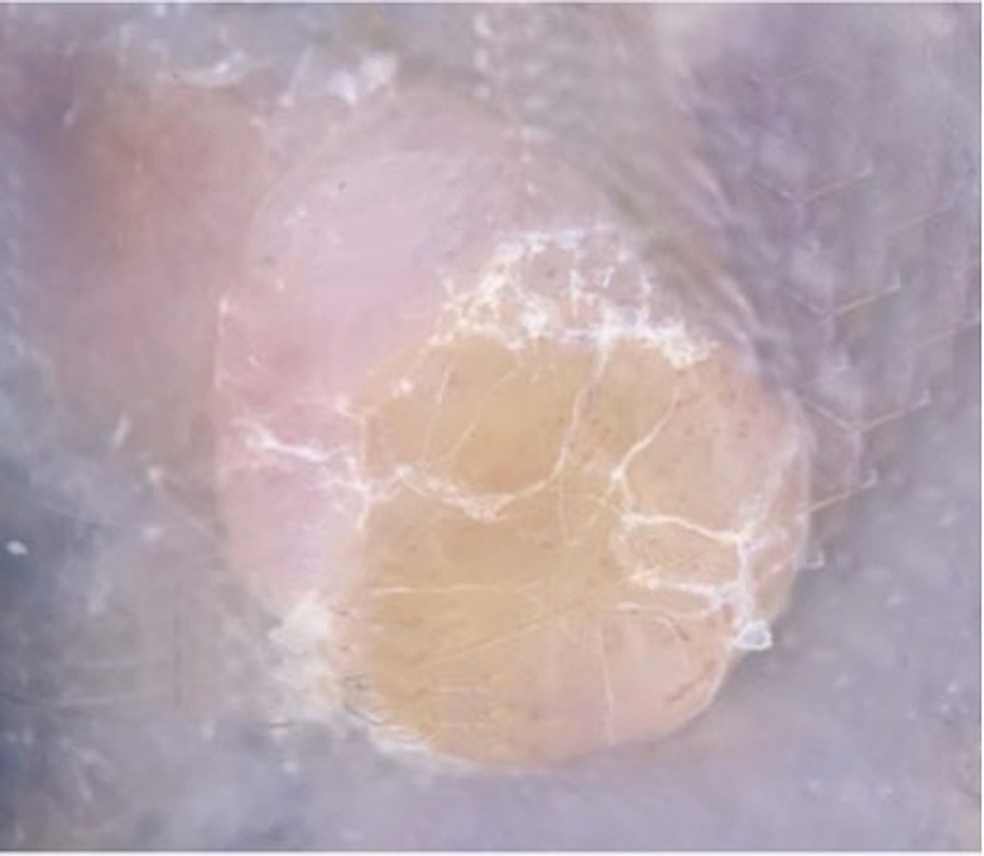
Dermoscopic visualization of a suspicious lesion on the right upper arm demonstrates an eccentrically located, structureless, pink to tan area with a homogeneous yellow-pink background, irregularly dotted and serpentine vessels, and overlying scale. Background tattoo pigment is also observed.

Dermoscopic examination revealed a structureless pink background with dotted vessels adjacent to a peripheral homogeneous yellow amorphous zone with irregularly dotted and serpentine vessels and overlying scale (Figure 2).

**Figure 3:**
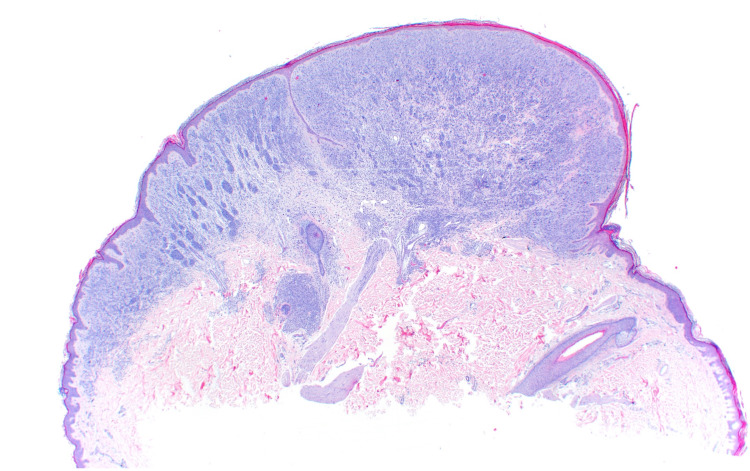
A low-power view highlighting the biphenotypic nature of the lesion, the eccentric zone of expansile growth compressing the surrounding epidermis is also noted here (H&E, x20). H&E: hematoxylin and eosin

**Figure 4:**
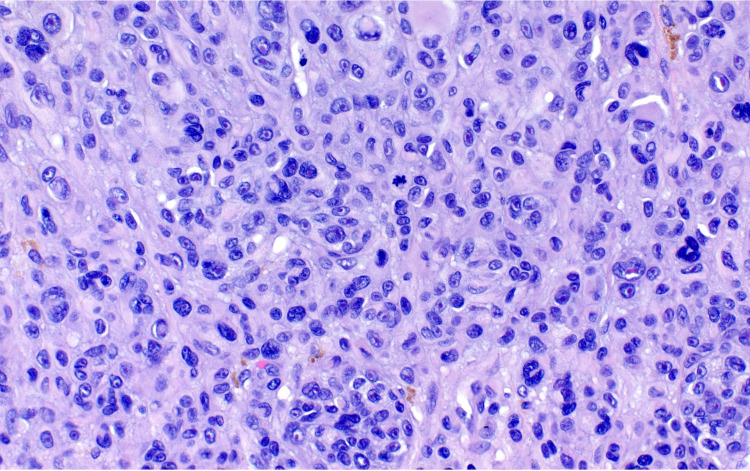
The expansile nodule demonstrates increased cellularity with ample eosinophilic glassy cytoplasm, moderate nuclear pleomorphism, and scattered dermal mitoses (H&E, x400). H&E: hematoxylin and eosin

A shave removal was performed. Histopathological examination revealed an atypical bi-phenotypic intradermal melanocytic proliferation with an eccentrically located expansile nodule compressing the surrounding epidermis, suggestive of rapid growth. The melanocytes within the expansile nodule displayed only slight cytologic atypia; a few mitotic figures were observed (Figures 3, 4).

**Figure 5:**
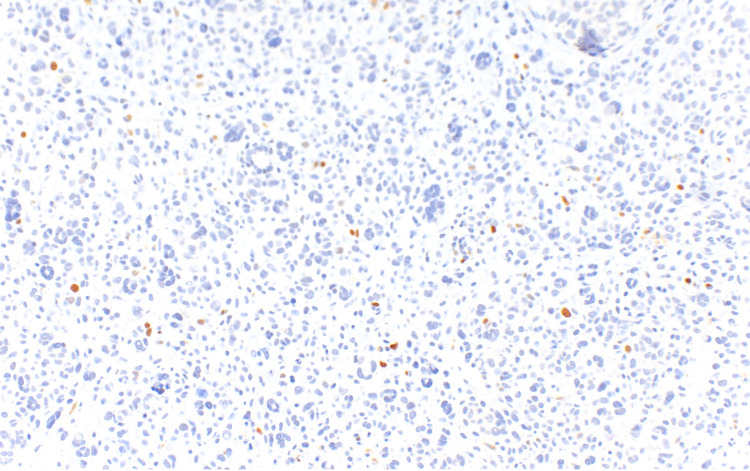
Immunohistochemical staining for Ki67 within the expansile zone (Ki67, x200). Ki67 - Proliferation marker protein

**Figure 6:**
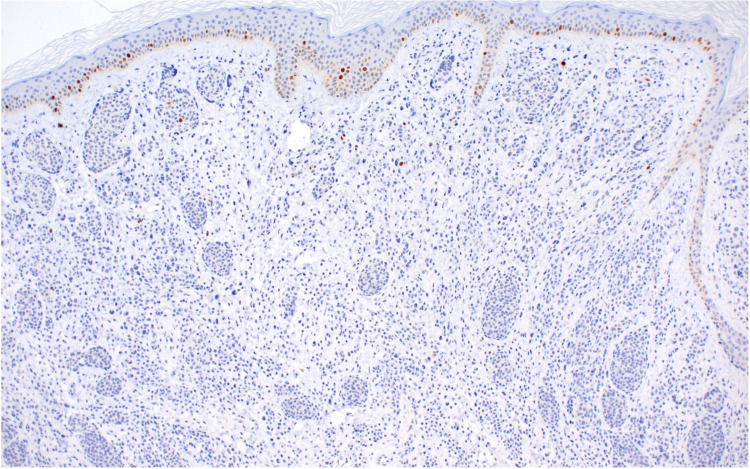
Immunohistochemical staining for Ki67 within the non-expansile zone (Ki67, x100) Ki67 - Proliferation marker protein

On immunohistochemistry, this lesion displayed a slightly increased Ki67 (a proliferation marker protein) index in the expansile zone (Figure 5) compared to the non-expansile zone (Figure 6).

**Figure 7:**
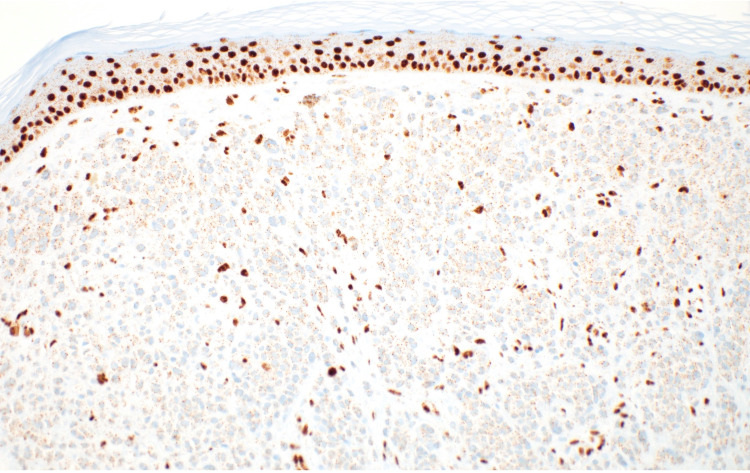
The tumorigenic nodule displays diffuse loss of BAP1 expression (BAP1, x200) BAP1: BRCA-Associated Protein 1

Diffuse loss of *BAP1* expression was observed (Figure 7).

**Figure 8:**
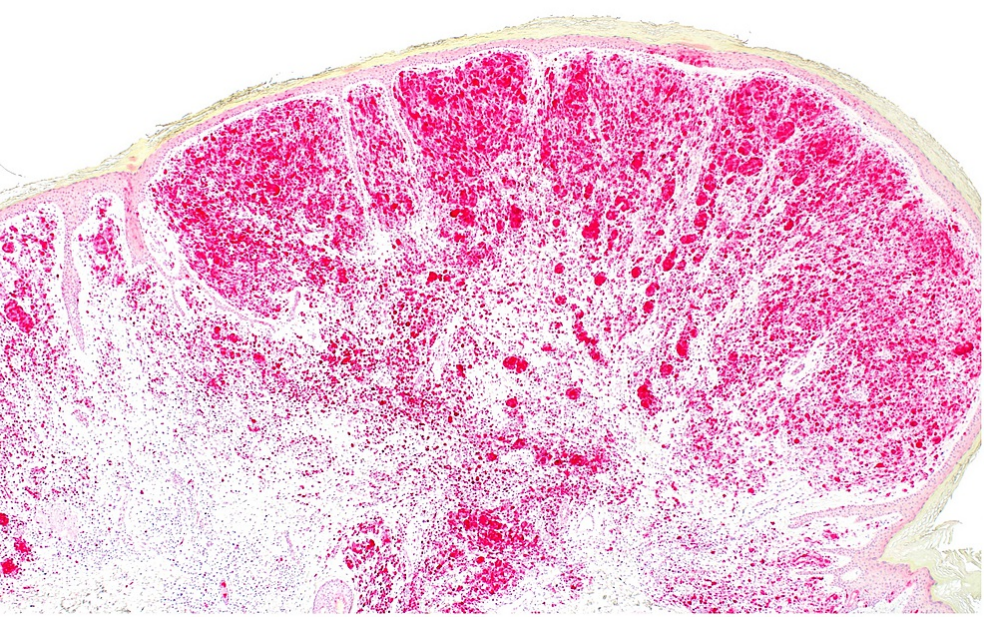
The tumorigenic nodule displays the expression of p16 (p16 x40). p16 - A tumor suppressor protein

Expression of p16 (a tumor suppressor protein) was diffusely retained (Figure 8). 

**Figure 9:**
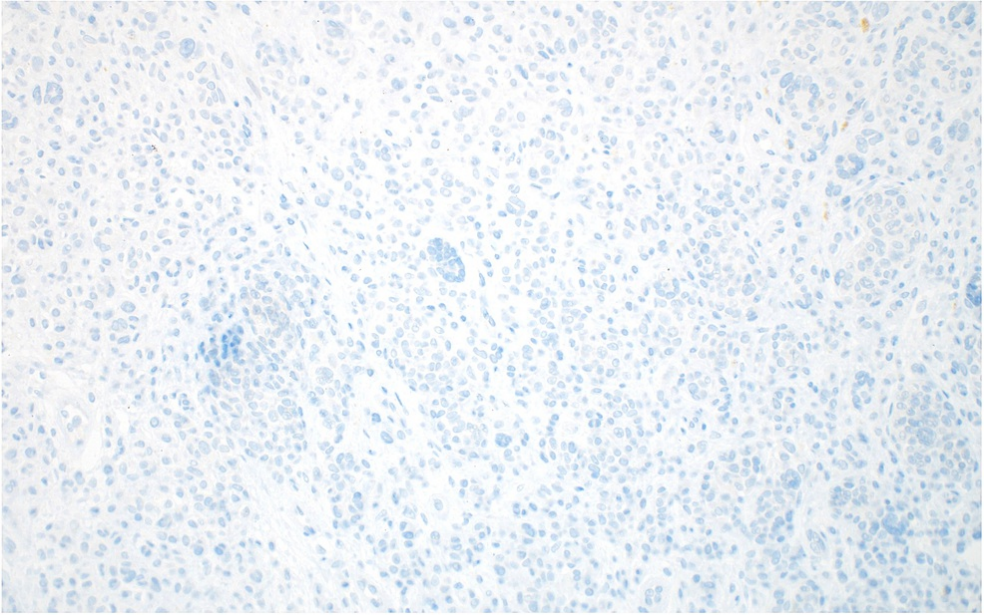
The tumorigenic nodule displays an absence of BRAF expression (BRAF, x200) *BRAF*: Human gene encoding the protein, B-Raf

*BRAF* (the gene that encodes a protein called B-Raf) expression was absent (Figure 9). 

Chromosomal microarray analysis was performed due to borderline histopathologic and immunophenotypic findings. Multiple abnormalities were observed in the expansile zone, including heterozygous copy number losses of entire chromosomes 3 and 9, losses of 8p and 11p, a 9 megabyte (Mb) segmental loss of 19q13.33q13.43, and a gain of 8q.

Though the histopathological findings were equivocal, considering the degree of chromosomal aberrancy, this lesion was deemed an atypical melanocytic lesion of uncertain malignant potential, and therapeutic management as an invasive melanoma was pursued. He underwent wide local excision, and a sentinel lymph node biopsy of the right axillary node was negative. The patient is currently being followed at six-month intervals without evidence of tumor recurrence or metastasis [4].

## Discussion

This patient’s cutaneous melanoma illustrates the unique dermoscopic and histopathologic findings of melanoma within a BAP1-inactivated tumor in a patient with BAP1-TPDS. While the *BAP1* gene was discovered in 2010, cutaneous BAP1-inactivated melanocytic tumors (BIMTs) were first described in 2011 by Wiesner et al. [5]. Sixty to seventy-five percent of patients with BAP1-TPDS present with BIMTs, including but not limited to Wiesner nevi, BAP1-inactivated nevi, atypical Spitz tumors, and BAP1-inactivated melanocytomas [6].

BAP1-TPDS diagnosis is determined by the identification of a heterozygous germline pathogenic variant in *BAP1* on molecular genetic testing [7]. BIMTs frequently manifest as well-demarcated, dome-shaped pink/tan to red/brown papules which commonly appear during the second and third decades of life [5,8]. Most patients with germline BAP1-TPDS present with between five to fifty tumors in any one of five proposed dermoscopic patterns. These tumors are, classically, intradermal proliferations of epithelioid melanocytes with glassy eosinophilic cytoplasm, occasional prominent lymphocytic infiltrate, and low mitotic activity [8]. 

The histopathologic findings of BIMTs are similar to Spitzoid nevi and yet are composed of one of two arrangements with cytologic atypia distinct from Spitz nevi [8]. They often demonstrate a prominent lymphocytic infiltrate and low mitotic activity [8]. The first of two arrangements, described as uniformly large epithelioid cells with distinct cytoplasmic borders and sheet-like growth, is commonly highlighted in lesions described as atypical Spitz nevi or nevoid melanomas containing *BAP1*-inactivating mutations [8]. The second pattern is biphasic and imitates tangential artifacts within lesions made up of small nevoid cells and large epithelioid cells [8]. In circumstances where molecular testing is inconclusive, authors have previously diagnosed melanomas in patients with BAP1-TPDS based on the presence of multiple dermal mitotic figures, genomic aberrations, and aggressive growth [5]. The histopathologic features of BAP1-inactivated tumors can overlap with melanoma; molecular studies help differentiate these lesions. Specifically, this patient’s lesion demonstrated an eccentric zone of sheet-like expansile growth, multiple dermal mitoses, and moderate pleomorphism. In a borderline lesion such as this patient’s, molecular analysis aids in clinical management.

Prior studies have characterized many of the molecular findings in malignant lesions in patients with BAP1-TPDS. Approximately 67% to 89% of cutaneous BIMTs simultaneously express BRAFV600E and *BAP1* gene mutations [8]. Curiously, melanomas found in patients with BAP1-TPDS typically lack BRAFV600E mutations [8]. *P16* gene expression is typically upregulated or retained, signaling the loss of other innate tumor suppressor genes within the cells that make up the lesion, as was the case in our patient’s lesion. This lesion had a heterozygous loss of chromosome 9, explaining the preservation of p16 staining by immunohistochemistry. Of note, tumorigenesis occurs on a pathway that does not directly involve *P16* expression in lesions with BAP1 mutations, and therefore p16 is not necessarily a reliable marker for prognostication in patients with BAP1-TPDS.

Five classic dermoscopic patterns have been identified in a cohort of patients with BIMTs [9]. However, the dermoscopic features of melanoma arising within BIMTs have not been previously described. Dermoscopy of this patient’s lesion revealed a peripheral homogeneous yellow amorphous zone with irregularly dotted and serpentine vessels and an overlying scale adjacent to a structureless pink zone with peripheral dotted vessels. Other dermoscopic features more frequently identified in suspected syndromic BIMTs include structureless pink/tan areas with atypical eccentric clods (with or without scale) that occasionally coalesce and multiple, interconnected, raised structureless areas that may be raised. We propose that the heterogeneous, biphasic pattern observed in our patient was a clue to the diagnosis of melanoma and predicted the biphenotypic histopathology.

Patients with BAP1-TPDS require increased surveillance, and BIMTs often share many similarities with - or may even harbor - unassuming malignant lesions, and distinguishing between benign and malignant lesions in this population may prove challenging. Clinical features such as rapid growth, in addition to heterogeneous clinical and dermoscopic morphologies, warrant biopsy in these patients. Histopathologic classification of borderline melanocytic tumors in the context of BAP1-TPDS is also challenging. In the absence of features of outright melanoma, the clinical presentation, dermoscopic features, histopathology, and molecular phenotypes of suspicious lesions must be used to evaluate the possibility of melanoma in patients with BAP1-TPDS.

## Conclusions

In summary, our report is the first to outline the specific dermoscopic findings of a BAP1 melanoma in a patient with BAP1-TPD. The dermoscopic findings clearly predicted the atypical biphenotypic histopathology, the borderline nature of which necessitated the use of chromosomal microarray analysis to support a diagnosis of melanoma.
